# Treatment, outcomes and characterization of pathogens in urinary tract infections caused by ESBL-producing Enterobacterales: a prospective multicentre study

**DOI:** 10.1093/jac/dkad402

**Published:** 2024-01-10

**Authors:** Hanna Montelin, Angela Camporeale, Anna Hallgren, Martin Angelin, Jonas Hogvall, Åse Östholm Balkhed, Malin Vading, Christian G Giske, Thomas Tängdén, the Swedish ESBL study group, Martin Angelin, Daniel Bremell, David Edenvik, Cecilia Eklund, Sofie Eriksson, Anna Hallgren, Helena Hallgren, Jonas Hansson, Thomas Hellborg, Hampus Hjorton, Jonas Hogvall, Maria Josephson, Julia Lenzen, Eva Lindqvist, Cecilia K Löfgren, Hanna Montelin, Pontus Nauclér, Maria Remén, Bo Settergren, Johan Tham, Malin Vading, Jon Wetterberg, Åse Östholm Balkhed

**Affiliations:** Department of Medical Sciences, Section of Infectious Diseases, Uppsala University, Uppsala, Sweden; Department of Laboratory Medicine, Division of Clinical Microbiology, Karolinska Institutet, Stockholm, Sweden; Department of Medical Sciences, Section of Infectious Diseases, Uppsala University, Uppsala, Sweden; Department of Clinical Microbiology, Infectious Diseases, Umeå University, Umeå, Sweden; Department of Infectious Diseases, Karlstad Central Hospital, Karlstad, Sweden; Department of Biomedical and Clinical Sciences, Division of Infectious Diseases, Linköping University, Linköping, Sweden; Department of Laboratory Medicine, Division of Clinical Microbiology, Karolinska Institutet, Stockholm, Sweden; Department of Infectious Diseases, Danderyds Hospital, Stockholm, Sweden; Department of Laboratory Medicine, Division of Clinical Microbiology, Karolinska Institutet, Stockholm, Sweden; Department of Clinical Microbiology, Karolinska University Hospital, Stockholm, Sweden; Department of Medical Sciences, Section of Infectious Diseases, Uppsala University, Uppsala, Sweden

## Abstract

**Objectives:**

Treatment options for urinary tract infections (UTIs) caused by ESBL-producing Enterobacterales are limited. Moreover, evidence to support therapeutic decisions is lacking. This study assessed current treatment strategies and patient and pathogen characteristics in relation to clinical and microbiological outcomes.

**Methods:**

Patients with UTI caused by ESBL-producing Enterobacterales were prospectively recruited by investigators at 15 infectious disease hospital departments. Data were collected on patient characteristics, treatments, clinical and microbiological cure 10–14 days after the end of treatment, and relapse within 3 months. Bacterial isolates were subjected to MIC determination and WGS.

**Results:**

In total, 235 patients (107 febrile UTI, 128 lower UTI) caused by *Escherichia coli* (*n* = 223) and *Klebsiella* spp. (*n* = 12) were included. Clinical and microbiological cure rates were 83% and 64% in febrile UTI, and 79% and 65% in lower UTI. Great variability in treatments was observed, especially in oral therapy for febrile UTI. No difference was seen in clinical outcomes with piperacillin/tazobactam (*n* = 28) compared with carbapenems (*n* = 41). Pivmecillinam was frequently used in lower UTI (*n* = 62), and was also associated with high clinical cure rates when used as initial therapy (10/10) or follow-up (7/8) for febrile UTI. Recurrent infection, diabetes mellitus and urogenital disease were associated (*P* < 0.05) with clinical failure and relapse. In *E. coli*, ST131 was significantly associated with relapse, and haemolysin with microbiological failure or relapse.

**Conclusions:**

Antibiotic treatments were highly variable. Patient and pathogen factors were identified as potential determinants of disease presentation and outcomes and may prove useful to guide individualized treatment and follow-up.

## Introduction

ESBL-producing Enterobacterales pose a major challenge in the management of urinary tract infections (UTIs).^1,2^ Evidence supports meropenem and imipenem for critically ill patients with bloodstream infections^3^ (BSIs) while IV administered β-lactam/β-lactamase inhibitor combinations (e.g. piperacillin/tazobactam and ampicillin/sulbactam) are often recommended in less severe cases with urinary source of infection.^4–6^

Finding narrow-spectrum and oral alternatives for these infections is a high priority for antimicrobial stewardship programmes to prevent accelerated emergence of resistance, particularly to carbapenems. However, oral treatment options are limited because of frequent co-resistance to commonly used antibiotics in ESBL-producing strains. Most isolates are resistant to ciprofloxacin and trimethoprim/sulphamethoxazole.^7^ Clinical data are scarce for other oral antibiotics of interest, such as pivmecillinam and amoxicillin/clavulanic acid. These antibiotics are, to some extent, active *in vitro* against ESBL-producing Enterobacterales and are sometimes used for lower as well as febrile UTI despite the lack of evidence.^8–10^

Previous studies suggest that genes encoding acquired resistance or virulence factors in the infecting pathogen may determine disease presentation and patient outcomes. For example, in *Escherichia coli* the virulence factor Iss was recently reported to be associated with severe sepsis^11^ and ST131 has been associated with worse clinical outcomes and relapse in UTI.^12–14^ A better understanding of the clinical importance of bacterial genetic markers could help guide pathogen-directed treatment.

This prospective multicentre observational study aimed to assess current treatment strategies and patient and pathogen characteristics in relation to outcomes in patients with UTI caused by ESBL-producing Enterobacterales across 15 hospitals in Sweden. Data were collected on antibiotic therapy, patient characteristics, and clinical and microbiological outcomes. Bacterial isolates were characterized by phenotypic antibiotic susceptibility testing and WGS to map resistance and virulence genes. Finally, we assessed patient and bacterial genetic determinants of clinical and microbiological outcomes.

## Materials and methods

### Study design

This observational study was conducted in collaboration with investigators at 15 infectious disease hospital departments. The local investigators prospectively identified patients that presented at the infectious disease department or upon consultation from other hospital departments or primary care centres. Inclusion criteria were: (i) age ≥18 years; (ii) significant growth (≥10^5^ cfu/mL) of ESBL-producing Enterobacterales in urine or growth in blood cultures; and (iii) at least one sign or symptom of UTI (i.e. fever, flank pain, dysuria, changes to urinary frequency or urgency). The classification of disease as febrile or lower UTI was made by the responsible physician based on clinical presentation and laboratory findings. Patients were included following written informed consent. Patients were excluded from the analysis if the bacterial isolates were resistant to all prescribed antibiotics or if more than one uropathogen was identified. The study was approved by the regional ethics committee in Uppsala (no. 2014/294), and recruitment was conducted from November 2014 to July 2017.

Participation in the study did not influence antibiotic therapy, which was prescribed at the discretion of the responsible physician. Therefore, recruitment was allowed at any timepoint during treatment. The first follow-up 10–14 days after completion of treatment (test of cure) included clinical assessment, documentation of medication intake and urine culture. The second follow-up performed 3 months after the end of treatment included assessment of relapse by phone consultancy and review of the medical records to identify recurrent UTI symptoms and repeated bacterial growth in urine.

### Data collection and follow-up

Clinical and microbiological data extracted from the medical records were transferred to an electronic case report form (eCRF, Pheedlt, version 3.03.016). Information was collected on patient demographics, comorbidities, risk factors for UTI (e.g. urogenital disease, urinary tract catheterization, diabetes mellitus; Table 1), previous UTI episodes, symptoms (e.g. fever ≥38.0°C, flank pain, dysuria; Table S1, available as Supplementary data at *JAC* Online), laboratory findings (C-reactive protein, WBC counts, S-creatinine) and antibiotic treatment.

### Definitions

Urogenital disease was defined as prostate cancer, benign prostate hypertrophy, bladder cancer, neurogenic bladder dysfunction, urinary tract anomaly, cervical cancer, urethral stone, recent (<6 months) urological procedure or renal transplantation. The infection was classified as complicated if at least one of the specified risk factors for UTI was present and as recurrent if the patient had ≥1 previous UTI in the past 6 months or ≥3 episodes of UTI in the past 12 months. The first administered antibiotic to which the pathogen was susceptible is referred to as initial treatment, and the last prescribed *in vitro* active antibiotic is referred to as definitive treatment. Clinical cure was defined as resolution of UTI symptoms, and microbiological cure as no bacterial growth or growth <10^3^ cfu/mL in urine sampled 10–14 days after the end of treatment. Relapse was defined as recurrent UTI symptoms within 3 months and significant growth in urine of the same bacterial species as reported in the initial episode.

### Phenotypic and genetic characterization of bacterial isolates

Antibiotic susceptibility testing was performed at the clinical microbiology laboratories. Isolates with resistance to cefotaxime and/or ceftazidime were subjected to ESBL testing with the double disc synergy method, as described in EUCAST guidelines for detection of resistance mechanisms. Bacterial isolates were collected for phenotypic and genetic characterization at the Department of Clinical Microbiology, Karolinska Institute, Stockholm, when possible. Antibiotics were purchased from Sigma–Aldrich (St Louis, MO, USA). The MIC values for a panel of clinically relevant antibiotics (Figure S1) were determined by broth microdilution using Sensititre DKMGN plates (Thermo Fisher Scientific, Waltham, MA, USA). Nitrofurantoin and mecillinam were tested with in-house microdilution and agar dilution, respectively. The results were interpreted according to EUCAST clinical breakpoints version 13.0.^15^ DNA was extracted by the MagNA Pure 96 System (F. Hoffmann-La Roche, Basel, Switzerland).

WGS was performed using the HiSeq platform (Illumina Inc., San Diego, CA, USA). Read quality, MLST and screening for resistance genes were assessed using microSALT (https://github.com/Clinical-Genomics/microSALT). Raw reads were analysed by the VirulenceFinder database^16^ to identify virulence genes in *E. coli*. After assembly with SPAdes (version 3.9),^17^ contigs were submitted to the BIGSdb database (https://bigsdb.pasteur.fr/klebsiella) to retrieve virulence genes in *Klebsiella* spp.

### Statistical analyses

All variables were compared to the rest of the study population (i.e. patients with versus without the variable of interest). For categorical variables, the chi-squared test with Yate’s correction and Fisher’s exact test for small counts were used as appropriate. The Mann–Whitney *U*-test was computed to compare continuous variables. A two-tailed *P *< 0.05 was considered significant. CIs were calculated using univariate logistic regression in IBM SPSS, version 27 (IBM, Armonk, NY, USA). Variables that showed significant associations with clinical or microbiological outcomes in univariate analysis were tested in multivariate logistic regression analyses.

## Results

### Patients and outcomes

A total of 247 patients were recruited. Of these, 12 patients were excluded due to lack of control culture (*n* = 7), growth of more than one bacterial species (*n* = 2) or resistance to all prescribed antibiotics (*n* = 3). The final analysis included 235 patients: 107 febrile UTI, of which 37 had BSI, and 128 lower UTI caused by *E. coli* (*n* = 223) or *Klebsiella* spp. (*n* = 12). Two febrile UTI patients had negative urine cultures (collected after initiation of treatment), but had bacterial growth in blood, fever and UTI symptoms. Almost half of the patients had medical conditions predisposing to UTI, and one-third of the episodes were classified as recurrent (Table 1). Clinical cure was achieved in 194/235 (83%) and microbiological cure in 151/235 (64%) of the patients (Table 2). Six patients were lost to follow-up after 3 months; relapse occurred in 34/229 (15%) of the evaluable cases. Outcomes were similar in patients with febrile UTI compared with lower UTI. No mortality was observed.

**Table 1.** Patient characteristics and risk factors for UTI

Variable	All *N* = 235	Febrile UTI *N* = 107	Lower UTI *N* = 128	*P* value
	*n* (%)			
Age (years), median (range)	64 (20–98)	63 (20–95)	65 (20–98)	NS
Sex				
Female	160 (68)	57 (53)	103 (81)	0.0001*
Male	75 (32)	50 (47)	25 (20)	
Healthcare setting				
Hospital	109 (46)	82 (77)	27 (21)	0.0001*
Primary care	126 (54)	25 (23)	101 (79)	
Risk factors				
Diabetes mellitus	44 (19)	28 (26)	16 (13)	0.01*
Urinary tract catheterization	28 (12)	17 (16)	11 (9)	NS
Indwelling urethral catheter	14 (6)	10 (9)	4 (3)	NS
Intermittent catheterization	12 (5)	5 (4.6)	7 (6)	NS
Suprapubic catheter	0 (0)	0 (0)	0 (0)	NS
Nephrostomy	2 (1)	2 (1.9)	0 (0)	NS
Urogenital disease^a^	72 (31)	30 (23)	42 (39)	0.01*
UTI frequency				
Recurrent UTI	71 (30)	34 (32)	37 (29)	NS
Sporadic UTI	162 (69)	73 (68)	89 (70)	
UTI complexity				
Complicated UTI^b^	106 (45)	60 (56)	46 (36)	0.003*
Uncomplicated UTI	129 (55)	47 (44)	82 (64)	

NS, not statistically significant.

^*^ *P *< 0.05, which was used as a cut-off for statistical significance. *P* values were calculated using the chi-squared test except for small observed values, where Fisher’s exact test was used.

^a^Prostate cancer, benign prostate hypertrophy, bladder cancer, neurogenic bladder dysfunction, urinary tract anomaly, cervical cancer, urethral stone, recent urological procedure or renal transplant.

^b^Diabetes mellitus, urinary tract catheterization or urogenital disease.

**Table 2.** Outcome in relation to disease presentation, patient factors, antibiotic treatment and infecting pathogen

Variable^a^	Clinical cure *N* = 235, *n* (%)	*P* value	Microbiological cure *N* = 235, *n* (%)	*P* value	Relapse *N* = 229^b^, *n* (%)	*P* value
All	194 (83)	NS	151 (64)	NS	34 (15)	NS
Febrile UTI, *n* = 107	89 (83)	NS	68 (64)	NS	15 (15)	NS
BSI, *n* = 37	31 (84)	NS	23 (62)	NS	8 (22)	NS
Lower UTI, *n* = 128	101 (79)	NS	83 (65)	NS	19 (15)	NS
Female, *n* = 160	130 (81)	NS	138 (86)	NS	24 (15)	NS
Male, *n* = 75	64 (85)	NS	58 (77)	NS	11 (16)	NS
Uncomplicated UTI, *n* = 129	110 (85)	NS	92 (71)	0.02*	18 (15)	NS
Complicated UTI, *n* = 106	84 (79)	NS	59 (56)	0.02*	17 (16)	NS
Urinary tract catheter, *n* = 28	22 (79)	NS	14 (50)	NS	7 (25)	NS
Urogenital disease, *n* = 70	55 (79)	NS	38 (54)	NS	19 (27)	0.002*
Diabetes mellitus, *n* = 44	37 (84)	NS	24 (55)	NS	12 (27)	0.03*
Recurrent UTI, *n* = 71	50 (70)	0.003*	57 (80)	NS	18 (26)	0.004*
Sporadic UTI, *n* = 162	142 (88)	0.003*	138 (85)	NS	16 (10)	0.004*
Initial IV treatment, febrile UTI, *n* = 70	58 (83)	NS	38 (54)	0.02*	11 (16)	NS
Definitive IV treatment, *n* = 28	24 (86)	NS	19 (68)	NS	3 (11)	NS
Definitive oral treatment, *n* = 42	34 (81)	NS	19 (42)	NS	8 (19)	NS
Initial oral treatment, febrile UTI, *n* = 37	31 (84)	NS	30 (81)	0.02*	4 (11)	NS
*E. coli*, *n* = 223	181 (81)	NS	146 (66)	NS	31 (14)	NS
*Klebsiella* spp., *n* = 12	9 (75)	NS	6 (50)	NS	3 (25)	NS

NS, not statistically significant.

^*^ *P *< 0.05, which was used as a cut-off for statistical significance. *P* values were calculated using the chi-squared test except for small observed values, where Fisher’s exact test was used.

^a^All variables were compared with the rest of the study population (e.g. patients with urinary tract catheters versus all other patients).

^b^Six patients were lost to follow-up after 3 months.

### Antibiotic treatment for febrile UTI

In patients with febrile UTI, 70/107 were initiated on IV antibiotics, most frequently a carbapenem (*n* = 41) or piperacillin/tazobactam (*n* = 28), administered as 30 min infusions. These antibiotics were always the first effective (*in vitro* active) agents and were often prescribed empirically (carbapenems, 30/41; piperacillin/tazobactam, 23/28). In the remaining cases, patients were typically started on cefotaxime and switched to a carbapenem or piperacillin/tazobactam within 2 days. The median duration of effective IV treatment was 5 days (IQR 4–11) and the median total duration of effective therapy was 11 days (IQR 10–14) (Table S1). There was no significant difference between the cohorts treated with a carbapenem compared with piperacillin/tazobactam in clinical (83% versus 82%) or microbiological (51% versus 57%) cure rates (Table 3). A higher proportion of BSI was noted in the carbapenem group (59% versus 43%); however, BSI was not associated with worse outcomes (Tables 2 and 3). In univariate and multivariate analyses, initial IV therapy was associated with a lower microbiological cure rate (38/70; 54%) compared with oral treatment (30/37; 81%) (Table 2).

**Table 3.** Antibiotic treatments and clinical and microbiological outcomes

Variable	Clinical cure	Microbiological cure	Relapse
	*n* (%)		
Patients with febrile UTI who were started on IV antibiotics			
* *Initial IV treatment, *n* = 70	58 (83)	38 (54)	11 (16)
* *Amikacin + colistin + tigecycline, *n* = 1	1 (100)	1 (100)	0 (0)
* *Carbapenem, *n* = 41	34 (83)	21 (51)	10 (24)
* * Ertapenem, *n* = 7	4 (57)	3 (43)	3 (43)
* * Imipenem, *n* = 7	7 (100)	3 (43)	1 (14)
* * Meropenem, *n* = 27	23 (85)	15 (56)	6 (22)
* * BSI, *n* = 24	20 (83)	14 (58)	4 (17)
* * Non-BSI, *n* = 17	14 (82)	7 (41)	6 (35)
* *Piperacillin/tazobactam, *n* = 28	23 (82)	16 (57)	1 (4)
* * BSI, *n* = 12	10 (83)	8 (67)	0 (0)
* * Non-BSI, *n* = 16	13 (81)	8 (50)	1 (6)
* *Definitive IV treatment, *n* = 28	24 (86)	19 (68)	3 (11)
* *Amikacin + colistin + tigecycline, *n* = 1	1 (100)	1 (100)	0 (0)
* *Ertapenem, *n* = 16	14 (88)	13 (81)	1 (6)
* *Imipenem, *n* = 1	1 (100)	0 (0)	0 (0)
* *Meropenem, *n* = 5	4 (80)	3 (60)	2 (40)
* *Piperacillin/tazobactam, *n* = 5	4 (80)	2 (40)	0 (0)
* *Definitive oral treatment, *n* = 42	34 (81)	19 (45)	8 (19)
* *Amoxicillin/clavulanic acid, *n* = 3	2 (67)	1 (33)	1 (33)
Amoxicillin/clavulanic acid + pivmecillinam, *n* = 2	2 (100)	0 (0)	0 (0)
* *Ceftibuten, *n* = 1	1 (100)	1 (100)	0 (0)
* *Ciprofloxacin, *n* = 15	11 (73)	8 (53)	3 (20)
* *Nitrofurantoin, *n* = 1	0 (0)	0 (0)	0 (0)
* *Pivmecillinam, *n* = 8	7 (88)	2 (25)	3 (36)
* *Pivmecillinam + fosfomycin, *n* = 2	2 (100)	1 (50)	0 (0)
* *Trimethoprim, *n* = 1	1 (100)	0 (0)	0 (0)
* *Trimethoprim/sulphamethoxazole, *n* = 9	8 (89)	6 (67)	1 (11)
Patients with febrile and lower UTI who were started on oral antibiotics			
* *Febrile UTI, *n* = 37	31 (84)	30 (81)	4 (11)
* *Ceftibuten, *n* = 3	3 (100)	2 (67)	0 (0)
* *Ciprofloxacin, *n* = 14	11 (79)	12 (86)	2 (14)
* *Mecillinam, *n* = 10	10 (100)	8 (80)	1 (10)
* *Moxifloxacin, *n* = 1	0 (0)	0 (0)	0 (0)
* *Nitrofurantoin, *n* = 5	3 (60)	4 (80)	1 (20)
* *Trimethoprim/sulphamethoxazole, *n* = 4	4 (100)	4 (100)	0 (0)
* *Lower UTI, *n* = 128	101 (79)	83 (65)	19 (15)
* *Ceftibuten, *n* = 4	2 (50)	1 (25)	0 (0)
* *Ciprofloxacin, *n* = 8	7 (88)	6 (75)	1 (13)
* *Fosfomycin, *n* = 3	2 (67)	1 (33)	1 (33)
* *Nitrofurantoin, *n* = 56	44 (79)	42 (75)	10 (18)
* *Pivmecillinam, *n* = 52	42 (81)	31 (60)	7 (13)
* *Trimethoprim, *n* = 2	1 (50)	1 (50)	0 (0)
* *Trimethoprim/sulphamethoxazole, *n* = 3	3 (100)	1 (33)	0 (0)

IV treatment was maintained as definitive therapy in 28 patients, while 42 patients were switched to oral antibiotics (Table 3). Ertapenem was associated with high clinical (14/16; 88%) and microbiological (13/16; 81%) cure rates. The median duration of treatment before shifting to an oral antibiotic was 4 days for carbapenems and 3 days for piperacillin/tazobactam. The median duration of oral follow-up was 8 days (IQR 7–10). Patients who were shifted to ciprofloxacin, trimethoprim/sulphamethoxazole or pivmecillinam had clinical cure rates of 73% (11/15), 89% (8/9) and 88% (7/8), respectively. Microbiological cure was less frequent, especially with pivmecillinam (2/8; 25%).

Thirty-seven patients received initial oral treatment, most commonly ciprofloxacin (*n* = 14) or pivmecillinam (*n* = 10) (Table 3). In the pivmecillinam group, all patients experienced clinical cure, eight were microbiologically cured and one patient relapsed within 3 months. The lower standard dose of 200 mg q8h was used in 8/10 cases, while 2 patients received 400 mg q8h, and the median treatment duration was 7 days (IQR 7–10).

### Antibiotic treatment for lower UTI

All patients with lower UTI received oral antibiotics (median duration 7 days, IQR 6–8). Pivmecillinam (*n* = 52) and nitrofurantoin (*n* = 56) were most frequently used and were associated with clinical cure rates of approximately 80% (Table 3). For pivmecillinam, clinical cure rates were 71% (22/31) in patients treated for ≤5 days and 86% (18/21) in patients treated >5 days; however, this difference was not statistically significant (*P *= 0.3). The lower standard dose of 200 mg q8h was used in 46/52 cases, while 6 patients received 400 mg q8h; no clinical failure or relapse occurred in patients who were prescribed the higher dose. In male patients, clinical cure was achieved in 7/10 cases treated with nitrofurantoin, 7/8 cases treated with pivmecillinam (200 mg q8h) and in all cases treated with ciprofloxacin (*n* = 3) or trimethoprim/sulphamethoxazole (*n* = 2).

### Delayed active treatment

Overall, 18% (43/235) of the patients did not receive effective (*in vitro* active) empirical antibiotic treatment. The median delay was 2 days (IQR 2–4 days), and this was not associated with worse outcomes; clinical cure was observed in 95% (41/43), microbiological cure in 72% (31/43) and relapse in 16% (7/43) of these cases.

### Associations between patient characteristics and disease presentation or outcome

In univariate analysis, febrile UTI showed significant associations with diabetes mellitus, urogenital disease, male sex and presentation in the hospital setting (Table 1). After adjusting for confounding variables in multivariate analysis, diabetes mellitus, male sex and presentation in the hospital setting demonstrated a persistent association (*P *< 0.001). In the univariate analysis, recurrent UTI (*P *= 0.003) was the only patient factor associated with clinical failure, while complicated UTI (*P *= 0.02) was associated with microbiological failure (Table 2). Relapse was associated with diabetes mellitus, urogenital disease and recurrent UTI, but only recurrent UTI maintained significance (*P *= 0.006) in the multivariate analysis.

### Antibiotic susceptibility of bacterial isolates

The clinical microbiology laboratories reported all isolates as MDR, i.e. non-susceptible to at least three antibiotic classes (Table S2). *E. coli* urine isolates were frequently resistant to ciprofloxacin (59%) and trimethoprim (63%), while susceptibility rates were high for mecillinam (95%) and nitrofurantoin (94%). MIC determination was performed for available isolates: 173/235 (74%) of the initial isolates and 22/86 (26%) of isolates detected during follow-up (Figure S1). According to the EUCAST clinical breakpoint for systemic infections (susceptible, S ≤ 8 mg/L), the amoxicillin/clavulanic acid susceptibility rate was 42% in *E. coli* urine isolates. When applying the clinical breakpoint for uncomplicated UTI (S 32 mg/L) in lower UTI cases, the susceptibility rate was 72%. Most *E. coli* isolates were susceptible to mecillinam according to the clinical breakpoint (S ≤ 8 mg/L), which applies to uncomplicated UTI. However, lower susceptibility rates were observed with agar dilution compared with disc diffusion, which is used in clinical routine (86% versus 95%). When applying the epidemiological cut-off value of ≤1 mg/L, which may be more appropriate for systemic infections, the susceptibility rate was only 36%. Strains isolated at follow-up invariably showed similar antibiotic susceptibilities as the initial strains.

### Genetic characterization

Genes encoding β-lactamases were found in 171/173 isolates: *bla*_CTX-M-15_ was most frequent (96/173; 55%), followed by *bla*_OXA-1_ (31%) and *bla*_TEM-1B_ (31%) (Tables S3 and S4). Genes encoding resistance to other antibiotics were commonly found: aminoglycosides (72%), fluoroquinolones (43%), sulphonamides (64%) and trimethoprim (61%). The *mcr-1* gene, associated with polymyxin resistance, was not identified in any of the strains. In *E. coli*, ST131 was observed in 44% (72/164) of the isolates, while *Klebsiella* spp. isolates were polyclonal. Toxins (haemolysin) were present in 21% of the *E. coli* isolates. All strains harboured at least one of the genes encoding adhesins and one gene belonging to the immune evasion and invasion categories. No additional acquired resistance was detected in strains isolated at follow-up compared with the initial strains.

### Associations between bacterial genetics and disease presentation or outcome

*E. coli* ST131 was associated with relapse in patients with febrile UTI (8/8, *P *= 0.002) (Table S4). C2, associated with CTX-M and fluoroquinolone resistance, was the predominant subclade in ST131 (52/72), and was more frequent in patients with febrile UTI (19/35) than lower UTI (45/129) (*P *= 0.05) (Table S5). Among the virulence families, toxins (haemolysin) were associated with lower microbiological cure rate rates in both febrile UTI (7/41, *P *= 0.04) and lower UTI (4/68, *P *= 0.0001), as well as relapse in febrile UTI (5/8, *P *= 0.03) (Table S4). No statistically significant associations between haemolysin or ST131 and outcome were identified in the multivariate logistic regression analysis. Due to the high prevalence of adhesins, it was not possible to determine their potential impact on disease presentation and outcomes.

## Discussion

This study provides data on the clinical management of UTI caused by ESBL-producing Enterobacterales across 15 hospitals in Sweden. The observational study design precludes definitive conclusions on the comparative efficacy of different antibiotic treatments. Still, the prospectively collected data on patients, outcomes and phenotypic and genetic characteristics of bacterial isolates add to the previously limited evidence. We observed large variability in the selection of treatments, which probably reflects the insufficient evidence and striving to use non-carbapenem and oral antibiotics where possible. Due to the lack of supporting data, many of the prescribed treatments are not recommended in national or European guidelines.^8,9^ Clinical cure rates were in line with what has been reported in other studies. However, microbiological failure was observed in more than one-third of the patients, which may be related to patient or pathogen factors, duration of treatment, dosing or an inability of the prescribed antibiotic therapies to eradicate the bacteria.

Carbapenems are proven effective and safe for severe infections caused by ESBL-producing Enterobacterales.^9^ In this study, initial treatment with a carbapenem or piperacillin/tazobactam was associated with comparable clinical and microbiological outcomes. We noted that 27/28 patients treated with piperacillin/tazobactam were prescribed the lower standard dose of 4.5 g q8h rather than 4.5 g q6h, which is recommended for ESBL-producing bacteria. Due to the observational design, carbapenems were likely used in more severe cases; yet, we found no difference in risk factors between the groups. Our findings align with previous studies that showed non-inferiority of β-lactam/β-lactamase inhibitor combinations compared with carbapenems in non-critically ill patients with urinary source of infection.^3–5,9^

Continued IV antibiotics as definitive treatment showed similar outcomes compared with oral follow-up. There was no difference in complicating factors or recurrent UTI between these treatment groups; however, there was likely a bias towards oral follow-up in patients who responded well to the initial therapy. Consistent with most other studies, the total treatment duration was normally 10–14 days (including 3–4 days of initial IV treatment) for febrile UTI and 7 days for lower UTI.^18^

Pivmecillinam has been proposed for febrile UTI, despite the lack of clinical evidence for this indication.^9,19,20^ However, the high susceptibility reported for mecillinam in ESBL-producing *E. coli* is valid only for uncomplicated UTI, and standard dosing (200–400 mg q8h) is insufficient to achieve adequate systemic exposures in relation to the current EUCAST breakpoints. The reference agar dilution method generated lower susceptibility rates than reported by the clinical microbiology laboratories based on disc diffusion, which indicates methodological difficulties. Adapted clinical breakpoints may be considered but would significantly lower susceptibility rates. Yet, pivmecillinam was used in 18 patients with febrile UTI and was associated with high clinical cure rates both alone (10/10) and when used as oral follow-up (7/8). A recent study reported clinical success in 88% of patients who received pivmecillinam 400 mg q6h as follow-up for *E. coli* (non-ESBL) BSI.^20^

The diversity of oral treatments for febrile UTI, including therapies with insufficient evidence and antibiotics only approved for lower UTI, illustrates the medical challenge in treating these infections. For instance, oral amoxicillin/clavulanic acid has only been reported to be effective for lower UTI caused by ESBL-producing *E. coli*^21^ and nitrofurantoin is not recommended for systemic infections because of subtherapeutic serum concentrations.^1^ To avoid misinterpretation, the microbiological laboratories must communicate which clinical breakpoint is applied for amoxicillin/clavulanic acid, because this has a significant impact on susceptibility (42% versus 72% in *E. coli* in this study), and that the reported susceptibility for nitrofurantoin (>90%) is valid only for lower UTI. Ciprofloxacin and trimethoprim/sulfamethoxazole remain drugs of choice against susceptible isolates and showed acceptable outcomes in this study. However, their use is hampered by co-resistance in most ESBL-producing isolates,^22^ and should be restricted to prevent further resistance development. Also, ciprofloxacin is advised only when there are no equivalent options due to the risk of long-lasting and irreversible side effects, according to EMA’s safety committee.^23^

Nitrofurantoin and pivmecillinam were the most frequently used antibiotics for lower UTI, and were associated with high clinical cure rates. These antibiotics are also first-line agents for lower UTI in male patients in Sweden, despite concerns regarding low tissue concentrations, and showed acceptable clinical efficacy in a previous study with non-ESBL-producing strains.^8,24^ Nitrofurantoin and oral fosfomycin, which is not marketed in Sweden, are highly active *in vitro* against ESBL-producing *E. coli* isolates and have been reported as successful for lower UTI.^25,26^ For pivmecillinam, observational and register-based studies show more conflicting results.^27–29^ Microbiological failure was common (60%) in this study but there was no increased risk of relapse, consistent with previous reports.^20^

Patients with complicating factors had a lower microbiological cure rate, and diabetes mellitus and urogenital disease were independently associated with relapse. Almost one-third of the patients had recurrent UTI, which was associated with a lower clinical cure rate and a higher risk of relapse. This observation agrees with previous studies and might reflect underlying patient factors that were not captured in our study.^9^ Research is warranted to explore if patients with recurrent UTI may benefit from higher dosing, prolonged treatment durations, or closer monitoring for treatment failure and relapse.

Moreover, we found associations between bacterial genetic features and clinical outcomes. ST131 in *E. coli* was more frequent in patients with febrile UTI who relapsed within 3 months, while the toxin haemolysin was associated with microbiological failure and relapse. *E. coli* ST131 typically carries genes encoding CTX-M-type ESBLs, which were also predominant in this study. Previous studies reported an association between ST131 and worse clinical outcomes, including 30 day mortality, treatment failure and relapse.^11,30–32^ Because WGS is becoming more frequently available to clinical microbiological laboratories, real-time genotypic assessment may soon be feasible to guide individualized therapy and follow-up based on pathogen characteristics.

The multicentre prospective design, including predefined assessment of clinical and microbiological outcomes, is a strength of this study. Unlike most previous studies, extensive genetic characterization of the causative bacteria was performed using WGS. We did not assess severity of illness, and treatments were only categorized as initial or definitive, which is a simplification of reality where several changes in therapy are often made and various dosing strategies and treatment durations are used. Observational data are always associated with risks of bias in treatment decisions and the sample size was too small to allow many subset or adjusted analyses. Nevertheless, this study provides new information on clinical practice in UTI caused by ESBL-producing Enterobacterales, including therapies that are not recommended in international treatment guidelines and for which evidence is limited, and may inform future randomized trials.

In conclusion, this study demonstrates significant diversity in antibiotic treatments for UTI caused by ESBL-producing Enterobacterales. Our results support the use of piperacillin/tazobactam in febrile UTI, while more research is needed to determine the efficacy of pivmecillinam and other oral antibiotics for this indication. Complicating patient factors, recurrent infection and bacterial genetic features (*E. coli* ST131 and haemolysin) showed associations with disease presentation and clinical or microbiological outcomes.

## Supplementary Material

dkad402_Supplementary_Data

## Local investigators of the Swedish ESBL study group

Martin Angelin (Umeå), Daniel Bremell (Göteborg), David Edenvik (Jönköping), Cecilia Eklund (Gävle), Sofie Eriksson (Västerås), Anna Hallgren (Uppsala), Helena Hallgren (Halmstad), Jonas Hansson (Luleå), Thomas Hellborg (Kalmar), Hampus Hjorton (Växjö), Jonas Hogvall (Karlstad), Maria Josephson (Helsingborg), Julia Lenzen (Skövde), Eva Lindqvist (Karlskrona), Cecilia K. Löfgren (Helsingborg), Hanna Montelin (Uppsala), Pontus Nauclér (Stockholm), Maria Remén (Eskilstuna), Bo Settergren (Kristianstad), Johan Tham (Malmö), Malin Vading (Stockholm), Jon Wetterberg (Gävle) and Åse Östholm Balkhed (Linköping).

## Supplementary data

Figure S1 and Tables S1 to S5 are available as Supplementary data at *JAC* Online.
