# Supplementary material for: Treatment, outcomes and characterization of pathogens in urinary tract infections caused by ESBL-producing Enterobacterales: a prospective multicentre study
Source: J Antimicrob Chemother. 2024 Jan 10;79(3):531–8. doi: 10.1093/jac/dkad402 (PMC10904723; doi:10.1093/jac/dkad402)
Supplement: dkad402_Supplementary_Data [file dkad402_supplementary_data.pdf]

Table S1. Symptoms, pathogens, and initiation and duration of antibiotic treatment.

Variable	All n=235	Febrile UTI n=107	Lower UTI n=128
	No. (%)		
**Disease presentation**			
UTI symptom	225 (96)	97 (90)	128 (100)
Flank pain	49 (21)	39 (36)	10 (8)
Lumbar tenderness	44 (19)	38 (35)	6 (5)
Nausea or vomiting	30 (13)	26 (24)	4 (3)
Micturition disorder	206 (87)	78 (72)	128 (100)
Dysuria	160 (68)	54 (50)	106 (83)
Urinary urgency	154 (65)	56 (52)	98 (77)
Increased frequency of urination	118 (50)	46 (43)	72 (56)
Fever (≥38.0°C)	87 (37)	85 (79)	2 (2)[a]
**Laboratory results**			
CRP, median; range	88; 1-491	117; 3-491	5;1-226
White blood cell counts, median; range	13; 4-29	14; 6-29	9; 4-22
Creatinine, median; range	102; 41-132	110; 43-39	69; 41-397
**Pathogens**			
Urine isolate			
E. coli	218 (95)	96 (92)	122 (97)
K. pneumoniae	11 (5)	7 (7)	4 (3)
K. oxytoca	1 (0.4)	1 (1)	0 (0)
Blood isolate			
E. coli	32 (86)	32 (86)	0 (0)
K. pneumoniae	4 (11)	4 (3)	0 (0)
K. oxytoca	1 (3)	1 (1)	0 (0)
**Antibiotic treatment**			
Days to effective treatment, median; IQR	0; 0–0	0; 0–2	0; 0-0
Days with effective treatment, median; IQR	8; 6-11	11; 10-14	7; 6-8
Days with effective IV treatment, median; IQR	5; 4-11	5; 4–11	0; 0
None	165 (70)	37 (35)	128
1–3 days	22 (9)	22 (21)	0
≥4 days	48 (20)	48 (45)	0
Days with active oral treatment, median; IQR	7; 6-10	10; 8-11	6; 6-8

[a]Two patients treated as lower UTI reported low-grade fever at home. These patients had symptoms consistent with lower UTI, were not febrile at the healthcare institutions, and had normal laboratory result.

Table S2. Antibiotic susceptibility profiles as reported from the clinical microbiology laboratories.

Antibiotic	*E. coli*								*Klebsiella* spp.							
	**Urine isolates**				**Blood isolates**				**Urine isolates**				**Blood isolates**			
	**No. of tested isolates**	**S**	**I**	**R**	**No. of tested isolates**	**S**	**I**	**R**	**No. of tested isolates**	**S**	**I**	**R**	**No. of tested isolates**	**S**	**I**	**R**
		**(%)**				**(%)**				**(%)**				**(%)**		
Amikacin	102	91	3	6	21	90	5	5	10	90	0	10	5	80	0	0
Amoxicillin/ clavulanic acid	85	40	0	60	3	33	0	67	7	14	0	86	0	0	0	0
Aztreonam	70	1	20	77	8	13	13	75	0	0	0	0	1	0	0	100
Cefadroxil	215	1	1	99	0	0	0	0	12	0	0	100	0	0	0	0
Cefotaxime	212	0	2	98	32	0	3	97	10	0	0	100	4	0	0	100
Ceftazidime	189	7	13	80	26	4	11	85	11	0	0	100	4	0	0	100
Ceftibuten	158	49	0	51	8	25	0	75	9	22	0	78	2	0	0	100
Ciprofloxacin	221	33	8	59	32	28	0	72	11	27	18	55	5	20	20	60
Ertapenem	54	98	2	0	15	100	0	0	6	100	0	0	1	100	0	0
Fosfomycin	18	89	0	11	1	100	0	0	7	86	0	14	0	0	0	0
Gentamicin	170	65	0	35	26	58	0	42	12	33	8	58	5	60	0	40
Imipenem	161	100	0	0	27	100	0	0	8	100	0	0	2	100	0	0
Mecillinam	217	95	0	5	2	100	0	0	12	75	0	25	1	100	0	0
Meropenem	196	100	0	0	29	100	0	0	12	92	8	0	4	100	0	0
Nitrofurantoin	217	94	0	6	0	0	0	0	6	0	0	100	0	0	0	0
Piperacillin/ tazobactam	204	74	15	11	31	55	19	26	11	46	36	18	5	40	20	40
Tobramycin	155	54	3	44	23	35	0	65	1	100	0	0	1	0	0	100
Trimethoprim	214	37	0	63	0	0	0	0	11	36	0	64	0	0	0	0
Trimethoprim/ sulphamethoxazole	198	37	0	63	30	37	0	63	11	46	0	55	5	40	0	60

Table S3. Genes encoding β-lactamases and resistance genes for other relevant antibiotics detected by WGS.

Resistance gene	**All** n=173	***E. coli*** n=164	***Klebsiella* spp.** n=9
	No. (%)		
bla$_{CTX-M}$	168 (97)	162 (99)	6 (67)
variant 15	96 (55)	90 (55)	6 (67)
variant 14	12 (7)	12 (7)	0 (0)
variant 27	32 (18)	23 (14)	0 (0)
other	20 (12)	20 (12)	0 (0)
bla$_{OXA-1}$	53 (31)	52 (32)	1 (11)
bla$_{TEM}$	58 (34)	55 (34)	3 (33)
variant 1B	53 (31)	50 (30)	3 (33)
other variants	5 (3)	5 (3)	0 (0)
bla$_{CMY-2}$*	5 (3)	5 (3)	0 (0)
bla$_{DHA-1}$*	3 (2)	3 (2)	0 (0)
other *bla* genes	20 (12)	12 (7)	8 (89)
**Aminoglycosides**	125 (72)	117 (71)	8 (89)
aac(3’)	53 (31)	48 (29)	5 (56)
aac(6’)lb-cr	52 (30)	51 (31)	1 (11)
aad	93 (54)	91 (55)	2 (22)
str	58 (34)	58 (35)	0 (0)
**Fluoroquinolones**	75 (43)	66 (40)	9 (100)
aac	56 (32)	52 (32)	4 (44)
oxqB	8 (5)	4 (2)	4 (44)
qnr	12 (7)	11 (7)	1 (11)
**Sulphonamide**	111 (64)	103 (63)	8 (89)
*sul*1	83 (48)	75 (45)	8 (89)
*sul*2	60 (34)	60 (36)	0 (0)
*sul*3	2 (1)	2 (1)	0 (0)
**Trimethoprim**	106 (61)	99 (60)	7 (78)

*Plasmid-mediated AmpC.

Table S4. Clinical and microbiological outcomes in relation to virulence factors and ST131 in *E. coli*.

Variable	Clinical cure No./no. of tested isolates (%)	*P* value	Microbiological cure No./no. of tested isolates (%)	*P* value	Relapse No./no. of tested isolates (%)	*P* value
**Febrile UTI**						
Adhesin, n=64	53/53 (100)	NS	41/41 (100)	NS	8/8 (100)	NS
Immune evasion, n=64	53/53 (100)	NS	41/41 (100)	NS	8/8 (100)	NS
Invasion, n=64	53/53 (100)	NS	41/41 (100)	NS	8/8 (100)	NS
Siderophore, n=63	52/53 (98)	NS	40/41 (98)	NS	8/8 (100)	NS
Proteases, n=49	40/53 (75)	NS	32/41 (78)	NS	7/8 (88)	NS
Toxins, n=17	14/53 (26)	NS	7/41 (17)	0.04*	5/8 (63)	0.03*
Other virulence factors, n=62	51/53 (96)	NS	40/41 (98)	NS	8/8 (100)	NS
Sum of virulence factors ≥6, n=50	41/53 (77.3)	NS	32/41 (78)	NS	8/8 (100)	NS
ST131, n=31	23/53 (43)	NS	17/41 (41)	NS	8/8 (100)	0.002*
**Lower UTI**						
Adhesin, n=100	80/80 (100)	NS	68/68 (100)	NS	16/16 (100)	NS
Immune evasion, n=100	80/80 (100)	NS	68/68 (100)	NS	16/16 (100)	NS
Invasion, n=100	80/80 (100)	NS	68/68 (100)	NS	16/16 (100)	NS
Siderophore, n=99	79/80 (99)	NS	67/68 (99)	NS	16/16 (100)	NS
Proteases, n=66	49/80 (61)	NS	44/68 (65)	NS	11/16 (69)	NS
Toxins, n=18	13/80 (16)	NS	4/68 (6)	0.0001*	2/16 (13)	NS
Other virulence factors, n=100	80/80 (100)	NS	68/68 (100)	NS	16/16 (100)	NS
Sum of virulence factors ≥6, n=66	49/80 (61)	NS	44/68 (65)	NS	11/16 (69)	NS
ST131, n=41	32/80 (40)	NS	27/68 (40)	NS	8/16 (50)	NS

*$P < 0.05$, which was used as a cut-off for statistical significance. *P* values were calculated using the chi-squared test except for small observed values, where Fisher's exact test was used.

Table S5. Results of clade analysis in *E. coli*.

Variable	**All** n=164	**Febrile UTI** n=64	**Lower UTI** n=100
	No. (%)		
Clade A (fim41)	10 (6)	3 (5)	7 (7)
Clade B	1 (1)	0 (0)	1 (1)
Clade C	52 (32)	23 (36)	29 (29)
Subclade C1	0 (0)	0 (0)	0 (0)
Subclade C2	35 (21)	19 (30)	16 (16)
Other C subclades	17 (10)	4 (6)	13 (13)
Other clades	9 (5)	5 (8)	4 (4)

Figure S1. MIC values as determined with broth microdilution and agar dilution (mecillinam) interpreted using EUCAST clinical breakpoints version 13.0.

a) *Escherichia coli*

**Antibiotic**	**No. of isolates with indicated MIC values (mg/L)**													
	≤0.03	0.06	0.12	0.25	0.5	1	2	4	8	16	32	64	128	>128
Amikacin								148	13	2	1	0		
Amoxicillin/clavulanic acid (lower UTI)[a]								24	20	15	13	12	16	
Amoxicillin/clavulanic acid (febrile UTI)[b]								8	19	7	12	10	8	
Aztreonam					4	3	10	21	31	36	33	26		
Cefotaxime					1	4	4	5	10	140				
Ceftazidime					11	13	18	36	38	33	15			
Ceftazidime/avibactam					162	1	0	0	1	0	0			
Ceftolozane/tazobactam					137	17	6	2	1	1	0	0		
Ciprofloxacin		38	7	22	9	1	0	87						
Colistin				131	31	1	0	0	0	1				
Ertapenem			155	6	3	0	0	0						
Gentamicin					81	27	3	2	0	51				
Imipenem					164	0	0	0	0	0	0			
Mecillinam (lower UTI)	0	0	0	5	14	21	18	10	8	6	8	2	8	
Mecillinam (febrile UTI)	0	0	0	1	7	15	13	6	14	1	0	2	5	
Meropenem			163	0	1	0	0	0	0	0	0			
Nitrofurantoin						0	0	0	2	14	48	16	12	7
Piperacillin/tazobactam						42	53	29	20	10	0	10		
Tigecycline				116	45	2	0	1	0					
Tobramycin						91	4	9	14	46				
Trimethoprim/sulfamethoxazole						68	0	0	0	94	2[c]			

Susceptible (MIC equal or below the tested concentration)
Susceptible
Susceptible, increased exposure
Resistant
Resistant (MIC beyond the tested concentration)

Concentration range
Max concentration range
[a] Breakpoint S≤32mg/L
[b] Breakpoint S≤8 mg/L
[c] Invalid results

b) *Klebsiella* spp.

Antibiotic	No. of isolates with indicated MIC values (mg/L)													
	≤0.03	0.06	0.12	0.25	0.5	1	2	4	8	16	32	64	128	>128
Amikacin								8	1	0	0	0		
Amoxicillin/clavulanic acid (lower UTI)[a]								0	0	0	1	0	1	
Amoxicillin/clavulanic acid (febrile UTI)[b]								2	3	1	0	0	1	
Aztreonam					2	2	0	0	0	0	4	1		
Cefotaxime					1	0	1	1	0	6				
Ceftazidime					2	0	0	0	1	4	2			
Ceftazidime/avibactam					7	0	0	0	0	0	1	1[c]		
Ceftolozane/tazobactam					6	2	0	0	0	0	0	1		
Ciprofloxacin		0	2	0	2	0	1	4						
Colistin				2	6	0	1	0	0	0				
Ertapenem			7	1	1	0	0	0						
Gentamicin					3	1	0	0	1	4				
Imipenem					8	0	0	1	0	0	0			
Mecillinam (lower UTI)	0	0	0	0	0	0	0	0	0	0	0	0	2	
Mecillinam (febrile UTI)	0	0	0	0	0	0	1	0	1	0	0	0	5	
Meropenem			8	0	1	0	0	0	0	0	0			
Nitrofurantoin						0	0	0	0	0	1	0	0	1
Piperacillin/tazobactam						1	1	3	3	0	0	1		
Tigecycline				3	3	2	0	1	0					
Tobramycin						3	2	1	1	2				
Trimethoprim/sulfamethoxazole						2	0	0	0	7				

Susceptible (MIC equal or below the tested concentration)
Susceptible
Susceptible, increased exposure
Resistant
Resistant (MIC beyond the tested concentration)

Concentration range
Max concentration range
[a] Breakpoint S≤32mg/L
[b] Breakpoint S≤8 mg/L
[c] Invalid results
